# Treatment of multiple synchronous canine mast cell tumours using intratumoural tigilanol tiglate

**DOI:** 10.3389/fvets.2022.1003165

**Published:** 2022-10-26

**Authors:** Graham K. Brown, Jessica R. Finlay, Rodney C. Straw, Joy Y. Ziea, Becky Leung, Kathleen O'Connell, Maurine J. Thomson, Justine E. Campbell, Pamela D. Jones, Paul Reddell

**Affiliations:** ^1^QBiotics Group Limited, Yungaburra, QLD, Australia; ^2^Perth Veterinary Specialists, Osborne Park, WA, Australia; ^3^Brisbane Veterinary Specialist Centre, The Australian Animal Cancer Foundation, Albany Creek, QLD, Australia; ^4^Animal Referral Hospital Brisbane, Sinnamon Park, QLD, Australia

**Keywords:** tigilanol tiglate, mast cell tumour, intratumoural, STELFONTA, multiple, synchronous, canine

## Abstract

Mast cell tumours (MCTs) are common canine skin neoplasia. While they generally occur as single tumours, multiple synchronous MCTs (msMCTs) of *de novo*/non-metastatic origin are reported in a proportion of the patient population. Where there is no evidence of metastasis or lymphatic spread, MCTs are effectively controlled by surgery and other local therapies. However, treatment of *de novo* msMCTs can be more challenging, especially when they occur in surgically difficult locations. Here, we report the use of tigilanol tiglate, a novel small molecule registered as a veterinary pharmaceutical for the local treatment of non-metastatic MCTs, in the treatment of patients with msMCTs presenting at three Australian specialist referral centres. We also present a meta-analysis of the literature to provide a better understanding of the prevalence of canine msMCTs. Notably, nine patients with a total of 32 MCTs were treated during the study. A complete response was recorded in 26 (81%) of the individual MCTs on Day 28 after a single tigilanol tiglate injection. Of the 6 initially non-responsive MCTs, one achieved a complete response after a further tigilanol tiglate treatment. A complete response was reported at 6 months in all 22 of the tumours that were evaluable and that had recorded a complete response at Day 84. For the literature meta-analysis, 22 studies were found with prevalence estimates of msMCTs ranging from 3 to 40%; when combined, these studies yielded 3,745 patients with a prevalence of 13% (95% CI 10; 16). Overall, the results demonstrate the utility of intratumoural tigilanol tiglate as an option for the treatment of multiple MCTs where multiple surgical resections would have been required.

## Introduction

Canine mast cell tumours (MCTs) are a common neoplasm primarily of the skin, and while most dogs are diagnosed with a single discrete lesion, closer inspection may reveal the presence of other local or distant lesions (1–16). Widely cited historical studies from the 1950s and 1960s suggest msMCTs have an occurrence rate between 11 and 14% in a total presenting population of just less than 400 MCT patients (12, 15–18). Patients with msMCTs most commonly present with *de novo* lesions, but each lesion may be of any grade and has the manifestation of local or distant invasion (Box 1) (3–16). The standard of care for patients presenting with msMCTs is to assess the grade of each lesion independently and stage the patient appropriately (1–16, 19–21). Where no evidence of metastasis or lymphatic spread is detected, most MCTs are effectively controlled with local therapy (1–16). Adjunctive therapies are considered when a patient presents with negative prognostic factors, for example, if a lesion is of high grade or exhibits negative molecular markers (1–16, 19–25).

Box 1 **Variable presentations of msMCTs.**

- 2–5 discrete Camus low-grade (Patnaik 1 or 2) lesions
  ° Local (≤ 10 cm apart) or distant (>10 cm apart)
- Many (>5) discrete Camus low-grade (Patnaik 1 or 2) lesions
- A mixture of high (Patnaik 3) and low-grade lesions
- Invasive primary high-grade lesion with multiple local satellite lesions (may be poorly differentiated)
- High-grade lesion with distant metastasis
- Local recurrence following inadequate treatment site control

Tigilanol tiglate is an approved veterinary pharmaceutical (STELFONTA^®^) for the intratumoural treatment of non-metastatic MCTs in Australia, the United States (US), the United Kingdom (UK), and the European Union. There are distinct differences in the specific label indications between the regulatory jurisdictions. The most significant is in relation to the resectable nature of the target MCT. The European Medicines Agency and the UK Veterinary Medicines Directorate both restrict tigilanol tiglate use to non-resectable MCTs. In contrast, the Australian Pesticides and Veterinary Medicines Authority and the US Food and Drug Administration have no label restriction on those tumours amenable to surgical excision and endorse a less conservative maximum treatable MCT volume of 10 cm^3^ or a maximum dose based on body weight of 0.25 mg tigilanol tiglate/kg (8 cm^3^ or 0.15 mg/kg on the EU/UK label) (26–28).

Tigilanol tiglate is a potent cellular signalling molecule with a multifactorial mode of action involving induction of a localised acute inflammatory response, immune cell recruitment to the treatment site, and disruption of tumour vasculature. At efficacious intratumoural doses, these processes cause haemorrhagic necrosis of the target tumour and its destruction results in the creation of a treatment site tissue deficit that is left unbandaged and generally heals uneventfully without the need for direct veterinary intervention (26–32). In a randomised, controlled, blinded clinical study of tigilanol tiglate for the treatment of canine MCTs carried out at 11 clinical sites in the US, treatment of a single MCT with tigilanol tiglate resulted in a complete response rate in 75% (60/80) of dogs (29). For dogs in that study that did not achieve a complete response with a single treatment, a second injection 30 days later increased the overall response rate to 88% (70/80 dogs) (29). A subsequent study of longer-term response durability in dogs from that trial found that after 12 months, 89% (57 out of 64) of evaluable patients were still recurrence-free at the treatment site (33).

Here, we expand on the findings of that US study to examine the efficacy of treatment with intratumoural tigilanol tiglate on a subset of patients with msMCTs enrolled in an Australian study of the drug on canine MCTs at four specialist referral centres during 2021 and 2022. We also systemically revisit the literature on the occurrence of msMCTs to undertake a meta-analysis aimed at providing a more contemporary perspective of the significance and prevalence of msMCTs.

## Materials and methods

### Case series

All patients that presented with msMCTs in a broader study of the use of tigilanol tiglate in the treatment of MCTs in a specialist referral setting in Australia were included in this case series. The study was conducted using protocols approved by the Queensland Department of Agriculture and Fisheries, Community Animal Ethics Committee (reference number CA 2020/11/1443), with written informed consent obtained from all owners prior to patient enrolment. Patients were required to satisfy eligibility criteria in relation to (a) general health, (b) evidence of metastatic disease (regional lymph node aspirates and abdominal ultrasound examination at the discretion of the treating specialist and consent of the owner), and (c) compliance with maximum label dose limits (a total dose rate of up to 0.25 ml/kg or no more than 5 ml per dog regardless of the number of tumours to be treated) (27–30). Fine needle aspirates of all target lesions were collected and submitted to Independent Veterinary Pathology (IVP, Australia) for cytological grading using the Camus system (34). Mandatory concomitant medications, comprising of a corticosteroid (prednisolone: 0.5 mg/kg b.i.d. for 7 days then s.i.d. for 3 days) to be started 2 days prior to treatment day and a H_1_ (chlorpheniramine: 0.25–0.5 mg/kg b.i.d. for 8 days) and a H_2_ (famotidine: 0.25–0.5 mg/kg b.i.d. for 8 days) histamine receptor blocker both to be started on treatment day, were dispensed, and a suitable pre-emptive analgesia plan formulated subsequent to enrolment (2, 35).

**Figure 1:**
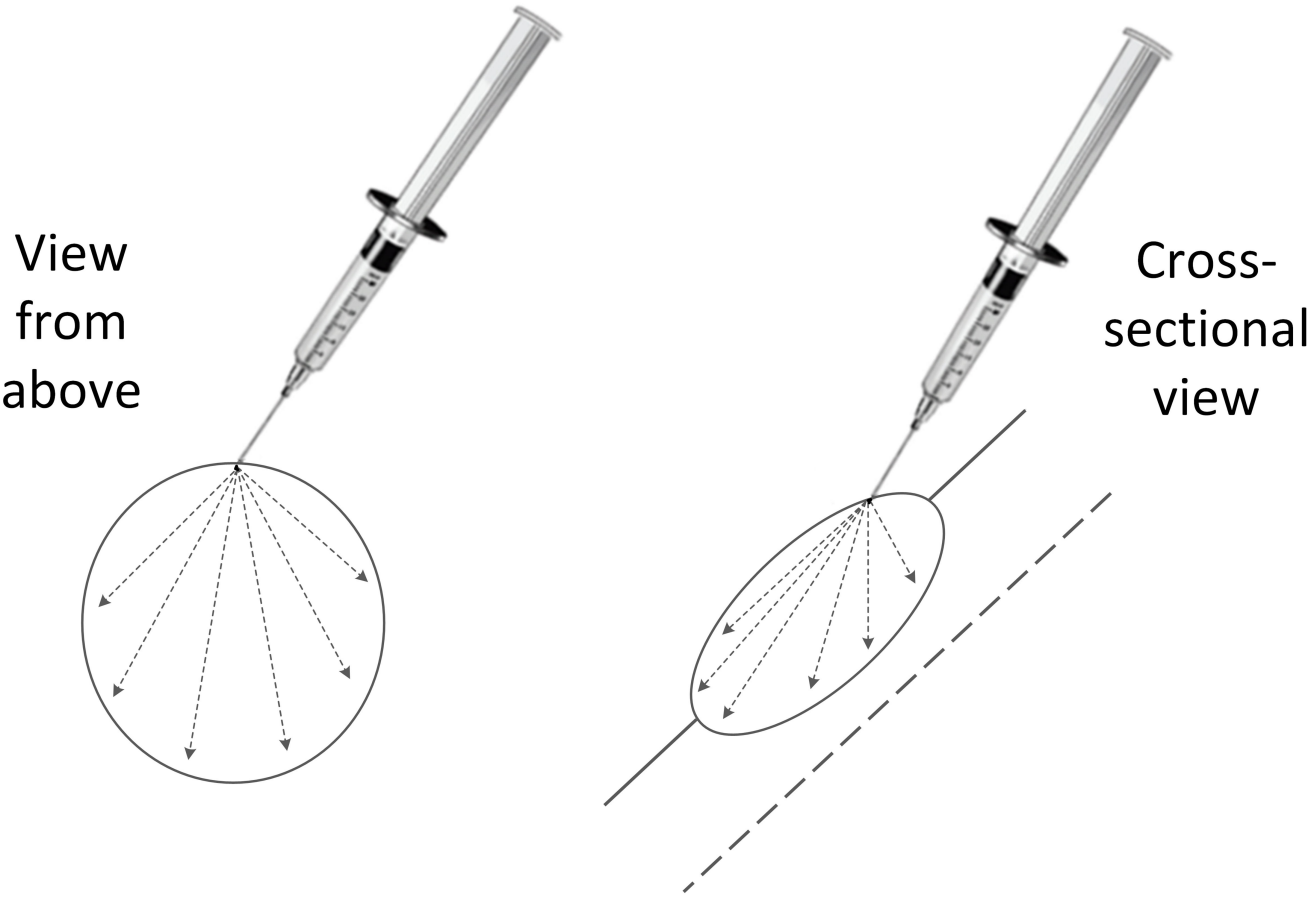
Fanning pattern used to disperse the intratumoural tigilanol tiglate dose throughout the tumour mass *via* a single injection point.

Tigilanol tiglate (1 mg/mL) is dosed by tumour volume. Dimensions of each tumour were measured with digital callipers, and then the volume(s) were calculated using a modified ellipsoid method where *tumour volume* = 0.5 × *length* (*cm*) × *width* (*cm*) × *depth* (*cm*) (36, 37). The dose per tumour to be administered is 50% of each tumour's volume (*tigilanol tiglate volume* = *tumour volume* × 0.5) and a minimum tumour dose of 0.1 ml (26–30). Personal protective equipment in the form of disposable gloves and protective eyewear were worn by the veterinarian and staff while the tigilanol tiglate dose was prepared and administered. Each patient was adequately restrained and where necessary, sedated to facilitate ease of administration. The dose for each tumour was drawn up into a separate Luer lock syringe with 23-26G ¾” needle attached. It was administered *via* a single injection point into each tumour and dispersed throughout the tumour mass by applying even pressure to the syringe plunger and moving the needle back and forth in a fanning pattern (Figure 1) (38).

Patient response was assessed in the clinic on Days 1, 7, and 28 after treatment, and owners were contacted by phone on Days 4 and 14. Treatment efficacy was assessed for each treated tumour on Day 28 and categorised using modified response evaluation criteria in solid tumours (RECIST) (39). Tumour response was classified based on changes in target tumour volume rather than the length of the longest tumour axis. Under this system, four categories of response in the target tumour were recognised: complete response (no evidence of remnant MCT), partial response (≥30% reduction in tumour volume), stable disease (< 30% change in tumour volume), and progressive disease (≥30% increase in tumour volume) (39). Adverse events were recorded and classified using the Veterinary Cooperative Oncology Group – common terminology criteria for adverse events (40). If a complete response was not achieved at Day 28, up to 2 retreatments could be carried out. Owners were contacted 3- and 6-months post-treatment to determine whether a treatment site recurrence had been observed, and an in-clinic assessment was recommended in the event of any noted treatment site changes by the owner. Those patients with a recurrence detected within 3 months were eligible for a re-treatment if enrolment criteria were still met.

### Systematic review and meta-analysis

A systematic review of the literature as of 31 January 2022, was undertaken using the databases CAB Abstracts *via* Web of Science (1910–present) and Medline *via* Ovid (1946–present) and the following search string: (mast cell OR mastocyt^*^) AND (tumo^*^ OR cance^*^ OR neoplas^*^) AND (canin^*^ OR dog OR dogs) NOT (review OR case). No limits were placed on geographic location or when the study was published. The same search terms were used in Google Scholar, and finally, a backward search was performed on reference lists from the relevant studies that had been gathered. Eligible studies had at least 80 enrolled participants and specifically reported on the number of them with at least two synchronous lesions.

The data collected was analysed and presented using R software (a metafor package used in conjunction with the tidyverse) (41, 42). Individual study prevalence estimates were logit transformed (log[p/(1–p)], where p = proportion); interstudy variance, *r*^2^, and pooled effect sizes were calculated using a random-effects model and the restricted maximum-likelihood estimator (REML method). Heterogeneity between studies was evaluated using Higgins *I*^2^ statistic, and a *post-hoc* subgroup analysis was performed to assess the effect of limiting the estimate to only include study populations greater than 150 or 200. A forest plot of the studies was created using the data gathered (43).

## Results

### Case series

A total of 32 tumours were treated during this case series on 9 eligible patients out of a total of 23 (39%) that participated in the larger MCT study. Brisbane Veterinary Specialist Centre and Perth Veterinary Specialists each had 4 patients, while Brisbane Animal Referral Hospital had a single patient. Signalment of the 9 patients in this case series is summarised in Table 1. Pre-treatment tumour characteristics along with case-series patient treatments are summarised in Tables 2, 3.

**Table 1:** Demographics of the nine case-series patients.

**Demographic**	**Value**
Median age in years on Day 0 (range)	7 (6–11)
Number of neutered females	6
Number of neutered males	3
Breed	
Staffordshire Bull Terrier	3
Pug	1
Labrador	3
Crossbreed	2
Median weight in kg (range)	32.8 (8.4–55.9)

**Table 2:** Summary of pre-treatment (Day 0) target MCT characteristics.

**Characteristic**	**Value**
Number consistent with low cytological grade (%)*	31 (97)
Median number detected (range)	2 (2–5)
Median maximum diameter in cm (range)	1.1 (0.4–2.9)
Median individual tumour volume in cm^3^ (range)	0.3 (0.01–6.8)
Median total volume per cycle in cm^3^ (range)	1.9 (0.2–9.9)
Median number treated per cycle (range)	2 (1–5)
Number of target MCTs located on:	
trunk (%)	18 (56)
limbs (%)	11 (34)
perineal (%)	2 (6)
head and neck (%)	1 (3)

* Cytologically graded using criteria described by Camus et al. (44).

**Table 3:** Treatment summary for each of the case-series patients.

**Identification**		**Location**	**MCT volume cm^3^ (tigilanol tiglate dose, mL)**	**Number of treatment cycles received**	**Single treatment RECIST classification**	**RECIST classification after up to three treatment cycles**		
**Patient**	**Individual MCT**				**Day 28**	**Day 28**	**Day 84**	**6 months**
02-02	A	Limb	1.5 (0.8)	2	CR	PR	PR	PR
	B	Trunk	0.3 (0.2)	1	CR	CR	CR	CR
03-01	A	Limb	0.3 (0.2)	2	SD	CR	CR	CR
	B	Trunk	0.3 (0.2)	3	SD	SD	N/A	N/A
03-09	A	Trunk	0.2 (0.1)	1	SD	SD	N/A	N/A
	B	Trunk	0.4 (0.2)	1	SD	SD	N/A	N/A
	C	Limb	1.1 (0.6)	1	PR	PR	N/A	N/A
	D	Limb	0.5 (0.3)	1	SD	SD	N/A	N/A
03-10	A	Perineal	0.03 (0.1)	1	CR	CR	CR	CR
	B	Limb	0.01 (0.1)	1	CR	CR	CR	CR
	C	Trunk	0.07 (0.1)	1	CR	CR	CR	CR
	D	Trunk	0.04 (0.1)	1	CR	CR	CR	CR
	E	Trunk	0.1 (0.1)	1	CR	CR	CR	CR
03-15	A	Trunk	1.7 (0.8)	1	CR	CR	CR	CR
	B	Trunk	1.2 (0.6)	1	CR	CR	CR	CR
	C	Limb	0.3 (0.2)	1	CR	CR	CR	CR
	D	Trunk	6.8 (3.4)	1	CR	CR	CR	CR
04-01	A	Trunk	0.3 (0.2)	1	CR	CR	CR	CR
	B	Trunk	0.1 (0.1)	1	CR	CR	CR	CR
	C	Limb	0.02 (0.1)	1	CR	CR	CR	CR
04-02	A	Trunk	1.1 (0.6)	1	CR	CR	CR	CR
	B	Trunk	1.6 (0.8)	1	CR	CR	CR	CR
	C	Limb	0.9 (0.5)	1	CR	CR	CR	CR
	D	Head	1.5 (0.8)	1	CR	CR	PR	PR
	E	Trunk	4 (2)	1	CR	CR	CR	CR
	F	Perineal	0.2 (0.1)	1	CR	CR	CR	CR
	G	Trunk	0.2 (0.1)	1	CR	CR	CR	CR
	H	Trunk	0.3 (0.2)	1	CR	CR	CR	CR
04-03	A	Limb	1.7 (0.9)	2	CR	PR	PR	PR
	B	Limb	0.1 (0.1)	1	CR	CR	CR	CR
04-08	A	Trunk	3.3 (1.7)	1	CR	CR	N/A	N/A
	B	Limb	6.6 (3.3)	1	CR	CR	N/A	N/A
Total number of CR (%)	26 (81)	25 (78)	22 (73)	22 (73)				

CR, complete response; PR, partial response; SD, stable disease; NA, not available for follow-up.

**Figure 2:**
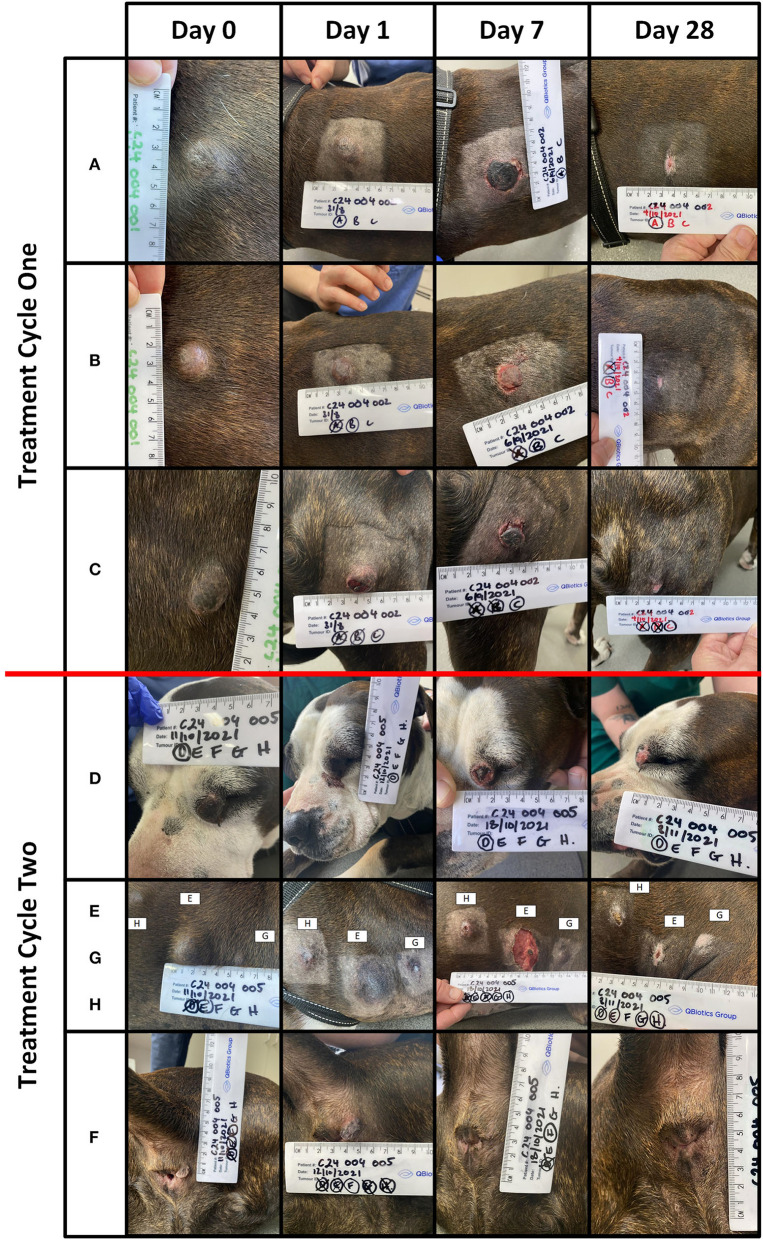
Patient 04-02 received two treatment cycles. Lesions **(A)** (volume = 1.1 cm^3^), **(B)** (volume = 1.6 cm^3^), and **(C)** (volume = 0.9 cm^3^) were treated in the first cycle. Lesions **(D)** (volume = 1.5 cm^3^), **(E)** (volume = 4 cm^3^), **(F)** (volume = 0.2 cm^3^), **(G)** (volume = 0.2 cm^3^), and **(H)** (volume = 0.3 cm^3^) treated in the second cycle. The images below are from Days 0, 1, 7, and 28, for lesions **(A–H)**.

**Figure 3:**
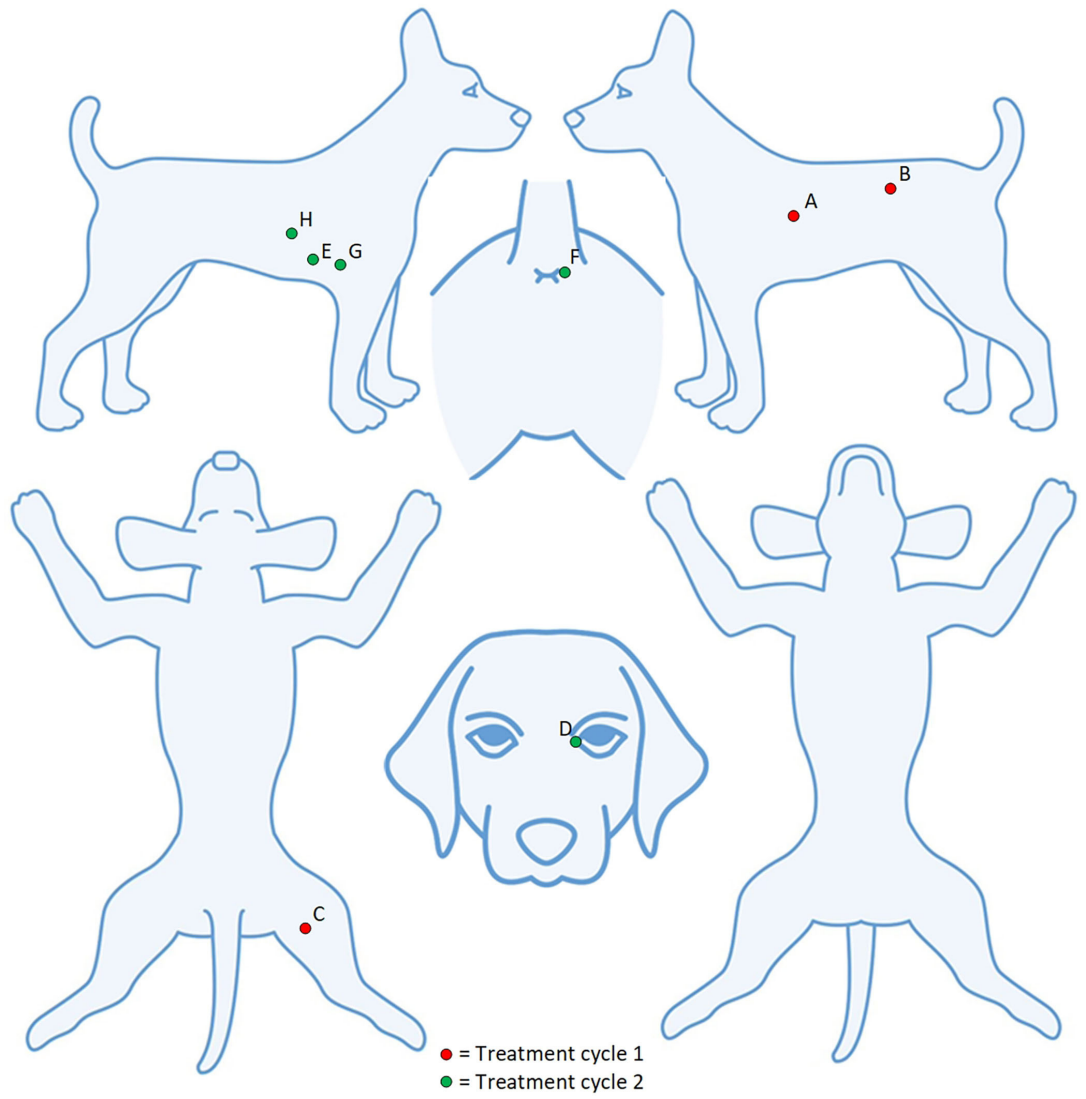
Patient 04–02 had eight tumours treated over two cycles. Five lesions (A–E) were detected at screening and three lesions (F–H) detected at the screening for the second treatment cycle.

Of the 32 tumours treated, 26 (81%) achieved a complete response by Day 28 with a single treatment and an additional tumour (27) after a second treatment. Three patients (03–10, 03–15, and 04–01) with a total of 12 tumours each achieved a complete response in all tumours treated, and the response was maintained at 6 months. Of the 9 patients, two patients (04–01 and 04–02) had new, distant (greater than 10 cm from the treatment site) lesions detected before Day 84. Patient 04–02 (Figures 2, 3) had three new MCTs included at the screening for a scheduled second treatment cycle.

**Figure 4:**
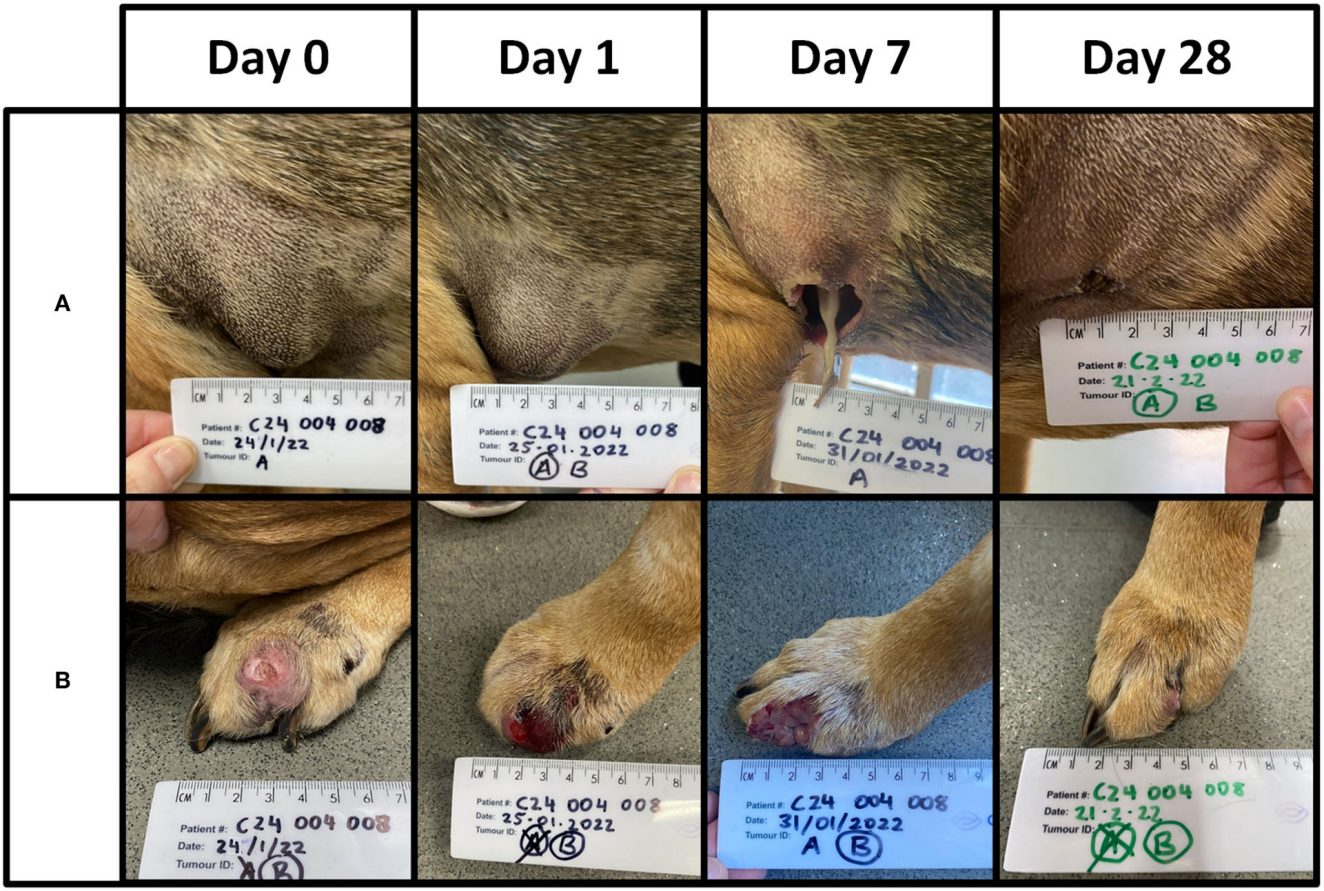
Patient 04-08 received a single treatment cycle for two lesions. Lesion **(A)** was of low grade and 3.3 cm^3^ in volume. Lesion **(B)** was of high grade and 6.6 cm^3^ in volume. A total dose of 5 mg (5 ml) was administered. The images below are from Days 0, 1, 7, and 28. A complete response was recorded for both lesions on Day 28. This patient was euthanised on Day 55 with no evidence of recurrence at site **(A)** or **(B)**.

Two patients (04–01 and 03–09) experienced adverse events within 28 days of treatment. Patient 04–01 developed hematemesis 6 days after treatment. The patient made a swift recovery, and the event, while serious, was determined by the attending specialist as likely unrelated to tigilanol tiglate due to the local nature of the treatment and the time that had elapsed. Patient 03–09 had four tumours treated and required an anaesthetic to attend to the treatment site of MCT B. The site had 10 ml of serosanguineous fluid aspirated from it and was subsequently flushed with saline. Three of the lesions were classified as stable disease, and one had a partial response. The owner opted against a second treatment cycle. One patient [04-08] had two lesions (Figure 4), one of which was consistent (met two out of four criteria) with a Camus (2016) high grade. This patient also had a soft tissue sarcoma unexpectedly diagnosed on Day 28 and was euthanised on Day 55 for reasons unrelated to any MCT.

Overall, there was a complete response rate of 73% (22 out of 30 tumours) after up to three injections per tumour at Day 84. Of the nine tumours that did not have a complete response to the first injection, five were recorded as stable disease. One of those (patient 03–01) received a second injection and achieved a complete response. Of the other 4 tumours, three recorded a complete response at Day 28, but a partial response was recorded at Day 84 due to the detection of recurrence at the treatment site. No new treatment site recurrences were recorded at 6 months.

### Prevalence of msMCTs meta-analysis

**Figure 5:**
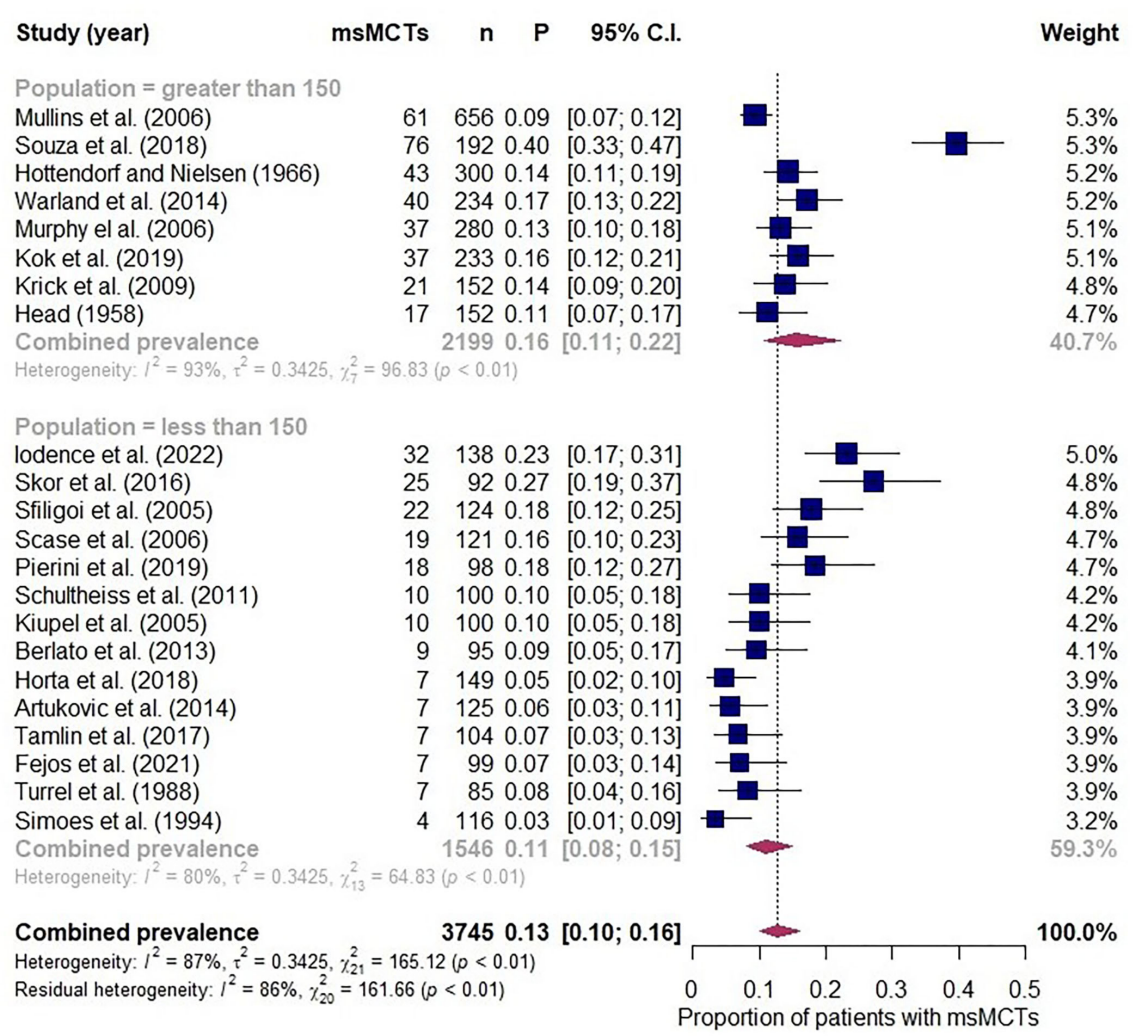
Forest plot depicting prevalence estimates from 22 independent observational studies of multiple synchronous mast cell tumours. Combined prevalence estimated along with a subgroup analysis assessing effect of excluding studies with less than 150 participants.

The database searches retrieved more than 800 results that were narrowed down to 22 meeting eligibility criteria (7–12, 17, 18, 34, 35, 45–56). A pooled population of 3,745 patients was analysed and, where reported, the median number of MCTs at presentation was 2 (range 2–6). Individual study prevalence of msMCTs at presentation ranged from 3 to 40% and a combined prevalence of 13% (95% CI 10–16%; Figure 5). Study heterogeneity was considerable but not unexpected with retrospective observational studies and the inclusion of multiple small studies sampling different populations over 5 continents published between 1958 and 2022. The subgroup analysis found comparably high heterogeneity between groups and minimal effect from excluding the highest and lowest outliers. The model was more robust with the inclusion of all 22 studies.

## Discussion

The case series included all enrolled patients who presented and were treated with msMCTs during a tigilanol tiglate efficacy and safety study from a selection of Australian oncology referral centres. Individual target tumour response rates in this series were generally comparable to those reported in the US pivotal efficacy and safety study. For example, following a single injection, there was a complete response in 81% of tumours on Day 28 in this study compared to a 75% response rate in the US study. Subsequent treatment of initially not fully responsive tumours increased the response rate to 84%, compared to 88% in the US trial. By Day 84, the complete response rate was 73% (22 out of 30 tumours), with all the tumours available for assessment at 6 months remaining tumour free. A finding consistent with Jones et al. (33) where a recurrence was most likely to occur within 6 months and largely by Day 84.

Local treatment site reactions observed in this study were associated with the mode of action of tigilanol tiglate. Tumour necrosis and slough resulted in the expected formation of a wound that resolved *via* secondary intention (Figures 2, 4). The time for wound formation, maximum wound size, and speed of wound healing in this study were comparable with the results from the published US study (29). In that study, eligibility criteria restricted investigators to the treatment of a single MCT, and the median patient dose on treatment day was 0.6 mL (median tumour volume of 1.1 cm^3^) (29). The case series investigated the treatment of msMCTs, of which the median individual tumour dose was less (median tumour volume of 0.3 cm^3^), but because multiple tumours could be sequentially treated on the same day, the combined median patient dose (1.0 mL) was higher. The total tigilanol tiglate dose (sum of individual target tumour doses treated on the day) must be within the label indication. To prevent this from being exceeded with the inclusion of all MCTs present, the highest priority lesion(s) should be targeted first and the remaining lesions treated at least 28 days later, on a subsequent treatment cycle (there is no limit to the allowed number of cycles). Likewise, on a distal limb, consideration should be given to target lesion proximity and the potential for two treatment sites to coalesce and form a single larger wound around the limb and extend healing time. This risk can be reduced by dividing lesions into separate treatment cycles.

There is a level of convenience with intratumoural tigilanol tiglate, but the mandatory concomitant medications require strict adherence to reduce the risk of degranulation. Prednisolone inhibits a signalling pathway that triggers mast cell degranulation, and in combination with H_1_ and H_2_ inhibitors that prevent histamine signalling, the risk of catastrophic, widespread mast cell degranulation is mitigated (57–62). Prednisolone has an additional, less predictable, effect on MCT volume (63–67). It downregulates the production of stem cell factor, a factor that both inhibits mast cell apoptosis and induces migration to the tumour as part of its mode of action (59, 66–69). Tumour volume is subject to variation following the 2 days of pre-treatment prednisolone. This may alter target tumour selection, so a degree of treatment day flexibility should be considered.

Canine MCTs are well reported in the literature, and two historical studies, Head (1958) and Hottendorf (1966), have commonly been referenced, 11 and 14%, respectively, for the prevalence of msMCTs (1, 12, 15, 16). Our meta-analysis was consistent with these historical estimates. However, this estimate is prone to bias as the focus of all but one of the included studies was not the presence of msMCTs. The observational studies included were retrospective analyses of medical records or pathology reports, and this may result in under-reporting. There may also be selection bias that favours their inclusion or exclusion from studies as their presence is a common study exclusion criterion (70–72). Another limitation was the lack of data on the primary or referral nature of the caseload for each prevalence estimate, and this precluded exclusion of studies where it was higher than expected. The lower limit of eighty participants was set to balance the generalisability of the prevalence estimate as smaller studies had a higher proportion of referral patients or were reporting on a particular treatment regimen with narrower eligibility criteria.

The propensity of certain individuals to develop many lesions over time can still make this neoplastic condition frustrating for the owner and clinician alike. In this case series alone, two patients would have required a second procedure within 6 months. Increased owner awareness can improve surveillance and early detection of lesions, enabling prompt treatment and mitigation of the risk posed by each new lesion. However, a level of owner fatigue may develop after an afflicted patient has had multiple surgical resections or an owner may fear further anaesthetics in an aging patient with increasing co-morbidities. Tigilanol tiglate offers a safe and efficacious therapeutic alternative to reduce the number of surgical procedures required and improve owner compliance where a non-surgical approach is favoured. Wound creation following intratumoural treatment can initially be confronting to both clinicians and patients without a prior understanding of the novel mode of action. However, healing of the wound site by secondary intention progresses in most cases in a highly predictable manner with a good functional and cosmetic outcome (31, 32, 73–75).

## Conclusion

This case series and meta-analysis shed light on the significance of msMCTs and illustrate the benefit of an intratumoural therapy in their treatment. The perceived occurrence of msMCTs is highly dependent on owner surveillance, their willingness at the time of discovery to get the lesion(s) assessed, and the ensuing thoroughness of the clinician's physical exam to search, find, and aspirate detectable masses. Surgery remains the current standard of care for local tumour control, but tigilanol tiglate should be considered as an efficacious alternative and dependent on patient compliance, can be administered in the consulting room or at least without a general anaesthetic. The utility and convenience of targeting new lesions as they arise ensure patients with a greater genetic propensity to develop MCTs are good candidates for this therapeutic option.

## Data availability statement

The original contributions presented in the study are included in the article/supplementary material, further inquiries can be directed to the corresponding author.

## Ethics statement

The animal study was reviewed and approved by the Queensland Department of Agriculture and Fisheries, Community Animal Ethics Committee. Written informed consent was obtained from the owners for the participation of their animals in this study.

## Author contributions

Independent specialist veterinary oncologists JF, RS, JZ, BL, KO'C, and MT enrolled the patients, delivered the treatments, and collected the clinical data. GB undertook the meta-analysis and prepared the first draft of the manuscript. All authors subsequently reviewed and revised. All authors contributed to the article and approved the submitted version.

## Funding

This work was funded by QBiotics Group Limited.

## Conflict of interest

Authors GB, JC, PJ, and PR are employed by QBiotics Group Limited. Authors JF, RS, JZ, BL, KO'C, and MT received funding to undertake the trial from QBiotics Group Limited. QBiotics Group Limited own the intellectual property, marketing authorisation and patents associated with tigilanol tiglate.

## Publisher's note

All claims expressed in this article are solely those of the authors and do not necessarily represent those of their affiliated organizations, or those of the publisher, the editors and the reviewers. Any product that may be evaluated in this article, or claim that may be made by its manufacturer, is not guaranteed or endorsed by the publisher.
